# Sensitivity to interaural level and time differences in individuals with autism spectrum disorder

**DOI:** 10.1038/s41598-022-23346-y

**Published:** 2022-11-09

**Authors:** Haruna Fujihira, Chihiro Itoi, Shigeto Furukawa, Nobumasa Kato, Makio Kashino

**Affiliations:** 1 NTT Communication Science Laboratories, 3-1 Morinosato Wakamiya, Atsugi, Kanagawa 243-0198 Japan; 2 Japan Society for the Promotion of Science, 5-3-1 Kojimahchi, Chiyoda-Ku, Tokyo, 102-0083 Japan; 3 Medical Institute of Developmental Disabilities Research, Showa University, Kitakarasuyama 6-11-11, Setagaya, Tokyo, 157-8577 Japan

**Keywords:** Autism spectrum disorders, Neurological disorders, Autism spectrum disorders, Psychiatric disorders

## Abstract

Individuals with autism spectrum disorders (ASD) are reported to exhibit degraded performance in sound localization. This study investigated whether the sensitivity to the interaural level differences (ILDs) and interaural time differences (ITDs), major cues for horizontal sound localization, are affected in ASD. Thresholds for discriminating the ILD and ITD were measured for adults with ASD and age- and IQ-matched controls in a lateralization experiment. Results show that the ASD group exhibited higher ILD and ITD thresholds than the control group. Moreover, there was a significant diversity of ITD sensitivity in the ASD group, and it contained a larger proportion of participants with poor ITD sensitivity than the control group. The current study suggests that deficits in relatively low-level processes in the auditory pathway are implicated in degraded performance of sound localization in individuals with ASD. The results are consistent with the structural abnormalities and great variability in the morphology in the brainstem reported by neuroanatomical studies of ASD.

## Introduction

Autism spectrum disorder (ASD) is diagnosed on the basis of impairments in social interaction and communication and restricted, repetitive behavioural patterns^1^. In addition to these symptoms, the majority of individuals with ASD have some degree of auditory dysfunction, such as auditory hypersensitivity^2–4^, auditory hyposensitivity^2,5^, and difficulty in speech understanding in noise^6,7^. These atypical responses to auditory stimuli are thought to be related to core diagnostic impairments in language and communication^8–11^.

Among the auditory dysfunctions in individuals with ASD, atypical sound localization is the focus of the current study. Sound localization—the ability to identify the direction of a sound source—is critical to the survival of a wide range of species^12^. Spatial hearing can help a person orient to a talker of interest in a crowded listening environment and thus contribute to human communication^13^. Difficulty in sound localization is considered to have implications for communication, the development of social behavior, and quality of life^11^. There is evidence that adults with ASD show degraded performance in vertical sound localization^14^. A study on event-related potentials has provided evidence of attenuated neural processing in the primary auditory cortex during a spatial discrimination task in adults with ASD^15^. Taken together, it is evident that individuals with ASD show degraded performance in sound localization. However, detailed characteristics of the localization problems have not been fully explored, and little information is available to elucidate its underlying mechanisms.

The subcortical auditory system plays a key role in sound localization^12,13^. In particular, neurons in the two principal nuclei of the superior olivary complex (SOC), the lateral and medial superior olives (LSO and MSO, respectively), are considered to process mainly interaural level differences (ILDs) and interaural time differences (ITDs), respectively, which are cues for horizontal sound localization^12^. Some studies have provided evidence of abnormalities in the human brainstem in individuals with ASD. Specifically, anatomical studies have shown a decreased number of neurons and neural dysmorphology in the human SOC in ASD^16–19^, and marked hypoplasia of the SOC and marked shortening of the pons in a woman with ASD^20^. In addition, magnetic resonance imaging (MRI) studies have shown a reduction in the pons in individuals with ASD compared with control individuals^21–23^. There is also a large number of studies on the auditory brainstem response (ABR) in individuals with ASD. The ABR to click sounds consists of seven positive peaks within 10 ms after the stimulus onset and whose waves with numbers from III up to and including wave V are considered to be generated in the auditory brainstem^24,25^. It has been reported that individuals with ASD showed prolonged wave III and V latencies compared to controls^26–37^. Recently, the amplitudes of the binaural interaction component (BIC) of the ABR, a component obtained by summing ABRs elicited by monaural clicks to each ear and subtracting this sum from the ABR elicited by binaural clicks and reported to arise primarily from neurons from the LSO^38,39^, have been reported to be significantly reduced in an ASD group compared to the control group^40^.

The neuroanatomical and neurophysiological evidence in individuals with ASD seems to implicate deficits in relatively low-level processes in the auditory pathway. It is possible that individuals with ASD have abnormal processing of ILD and ITD cues, leading to degraded performance in sound localization. Lodhia and colleagues reported that adults with ASD showed atypical event-related potentials (e.g., object-related negativity and P400) to stimulus that were manipulated in ILD and ITD^41,42^. However, to our current knowledge, no study has provided direct evidence that individuals with ASD have abnormal processing of ILD and ITD cues.

The present study aimed to examine whether lower level processes of auditory cues, specifically ILD and ITD, are implicated in degraded performance of sound localization in individuals with ASD. We measured the psychophysical sensitivity to ILD and ITD cues using a sound lateralization task. Our hypothesis was that individuals with ASD would show lower sensitivity to the ILD and/or ITD cues than controls. The present study also compared the diversity of the lateralization performance between ILD and ITD. Some studies on auditory perception (e.g. frequency discrimination) have suggested the existence of a subgroup of abnormal discriminators in ASD groups^9,43,44^. Furthermore, anatomical studies have reported a great variability in the morphology of the MSO neurons in ASD^16–18^. It is possible that the heterogeneity of the ASD population could also be observed in the performance for a particular sound-localization cue (e.g., ITD). The results overall should provide insights into not only the mechanisms behind sound-localization deficits, but also the prevalence of heterogeneity across auditory processes in ASD individuals.

## Results

Participants with ASD and controls were statistically matched for age and IQ score (shown in Table 1). All participants had normal hearing thresholds (≤ 25 dB HL from 125 to 8000 Hz). The frequency selectivity for each ear also was assessed by measuring the bandwidth of auditory filters [in equivalent rectangular bandwidth (ERB)] centered at 500, 1000, and 2000 Hz^45,46^. Cochlear hearing loss is often accompanied by broadened auditory filters^47,48^. The ERBs obtained from the ASD and control groups are summarized in Table 2. No significant difference between the two groups was found at any of the center frequencies. That is, there was neither an indication that the participants in the present study had cochlear hearing loss nor that the auditory sensitivities attributed to individual ears were significantly different between the ASD and control groups.

**Table 1:** Demographic information of the ASD and control groups (shown as mean ± standard deviation).

	ASD	Control	t-test
Gender (female:male)	4:25	11:26	–
Age	29.6 ± 6.4	28.5 ± 5.0	0.44
Full Scale IQ	104.7 ± 12.9	108.3 ± 14.5	0.31
AQ Score	35.0 ± 6.7	18.8 ± 5.1	< 0.001

**Table 2:** Summary of statistics of ERBs (in hertz) for ASD and control groups at each center frequency.

	ASD		Control		Student’s t-test		
	Mean	SD	Mean	SD	*t*	*df*	*p*
500 Hz (A: *n* = 12, C: *n* = 17)	100.5	28.4	88.1	14.9	1.535	27	0.137
1000 Hz (A: *n* = 17, C: *n* = 20)	137.5	21.1	141.4	23.5	− 0.528	35	0.601
2000 Hz (A: *n* = 12, C: *n* = 17)	257.9	66.4	304.5	77.8	− 1.685	27	0.104

**Figure 1:**
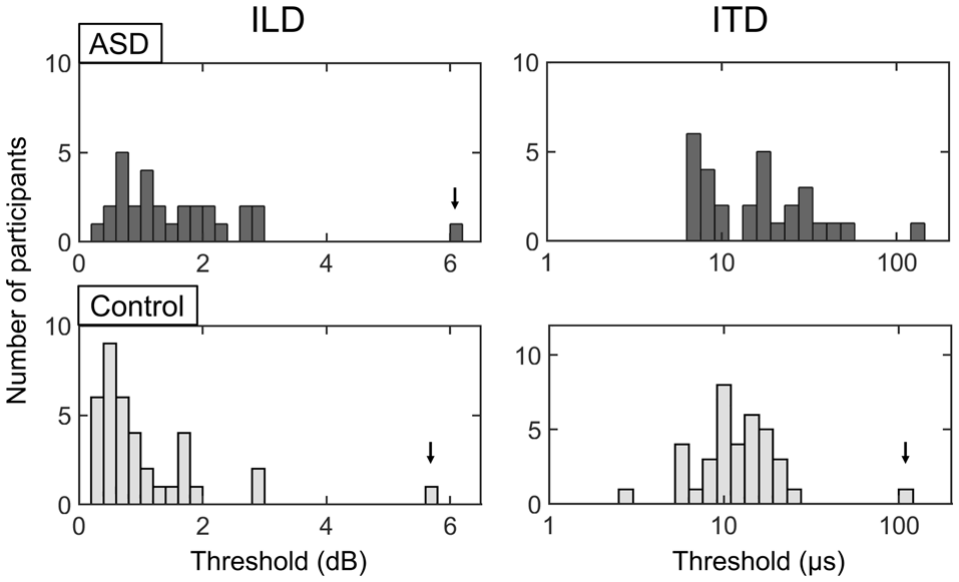
Histograms of ILD and ITD thresholds in ASD and control groups. The arrows indicate outliers.

Histograms of the ILD and ITD thresholds for the ASD and control groups are shown in Fig. 1. Some outlying scores were observed in the ASD and control groups (ILD: *n* = 1 for ASD; *n* = 1 for control. ITD: *n* = 1 for control). These outlying scores were above 3 standard deviations (SDs) relative to the means. Levene's test for equality of variance indicated a significant difference in variances of the ITD thresholds between the ASD and control groups for outlier-excluded data (*p* = 0.008), but did not reach the statistical significance for outlier-included data (*p* = 0.072). There were no significant differences in variances of the ILD thresholds between the two groups (*p* = 0.362 with outliers included; *p* = 0.114 with outliers excluded). Kolmogorov–Smirnov test for outliers excluded data showed normal distributions for the ITD thresholds (ASD: *p* = 0.020, control: *p* = 0.020), but not for the ILD thresholds in the control group (ASD: *p* = 0.170, control: *p* = 0.001). Therefore, the non-parametric test (Mann–Whitney U test) and parametric test (Welch’s t-test) were used for comparing the central tendency between groups (ASD vs. control) for the ILD and ITD thresholds, respectively. Table 3 shows the medians, the quartile deviations (QDs), and *p*-values related to the Mann–Whitney U test results for the ILD thresholds. The Mann–Whitney U test showed that the ASD adults had significantly higher ILD thresholds than the control adults (*z* = − 2.823, *p* = 0.005). Table 4 shows the means, the SDs, and *p*-values related to the Welch’s t-test results for the ITD thresholds (outliers excluded). The ASD adults showed significantly higher ITD thresholds than the control adults (*t* = 2.274, *p* = 0.028). These results indicate that participants with ASD exhibited poorer ILD and ITD sensitivity than the controls.

**Table 3:** Medians, quartile deviations, and *p*-values related to Mann–Whitney U test for the ILD thresholds.

	ASD		Control		Mann–Whitney U test
	Median	QD	Median	QD	*p*
ILD threshold (dB) (A: *n* = 29, C: *n* = 37)	1.25	0.67	0.66	0.41	0.005

**Table 4:** Means, SDs, and *p*-values related to Welch’s t-test for the log-transformed ITD thresholds (outliers excluded).

	ASD		Control		Welch’s t-test
	Mean	SD	Mean	SD	*p*
Log ITD threshold (Units) (A: *n* = 29, C: *n* = 36)	1.21	0.33	1.05	0.20	0.028

There was a significant diversity in participants with ASD for the ITD thresholds as shown in the results of the Levene's test. These observations are in line with the notion that an ASD group can consist of subgroups with normal and abnormal performance, as reported by Jones et al.^43^, who showed the existence of an ASD subgroup with “exceptional” auditory skills. Following Jones et al.^43^, we defined subgroups of poor and good performers as those exhibiting thresholds that were above and below 2 SDs, respectively, relative to the mean of the control group (outliers excluded). Seven adults in the ASD group (24.1% of the ASD group) demonstrated high ITD thresholds (i.e., poor performers), while only one adult in the control group (2.7% of the control group) fell in that subgroup. This difference is statistically significant (Fisher’s exact test, *p* = 0.018), indicating that the ASD group was significantly associated with high ITD thresholds. There were no good performers (i.e., low threshold) in the ASD group, while one adult in the control group (2.7% of the control group) fell in that subgroup. There was no significant difference in low ITD thresholds between the ASD and control groups (*p* = 1.000, Fisher's exact test). Taken together, there was a significant diversity of ITD sensitivity in the ASD group, and it contained a larger proportion of participants with poor ITD sensitivity than the control group.

As in the analyses for ITD, we defined poor and good performers in terms of ILD thresholds, based on the mean and SD for the control group. There were no significant differences between the ASD and control groups in high ILD thresholds (20.7% of the ASD group, 8.1% of the control group; *p* = 0.166) or low thresholds (no applicable participant).

**Figure 2:**
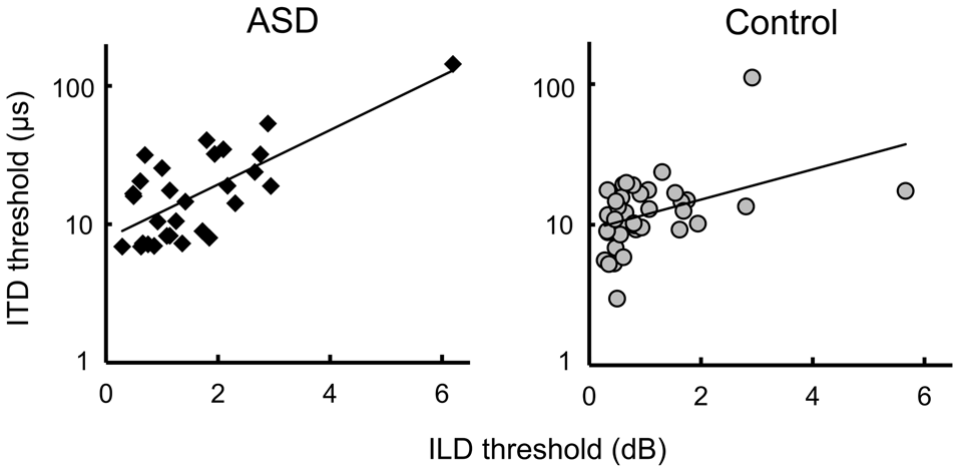
Scatter plots of ILD thresholds versus ITD thresholds in ASD group (left) and control group (right).

We also examined the relationship between the ILD and ITD thresholds using Spearman’s correlation coefficients (*r*_*s*_) across data from the ASD and control groups. There were significant correlations between ILD and ITD thresholds in the ASD group (*r*_*s*_ = 0.555, *p* = 0.002) and the control group (*r*_*s*_ = 0.471, *p* = 0.003). Figure 2 shows the relationship between the ILD and ITD thresholds. The correlations were significant even though the outliers were excluded (ASD: *r*_*s*_ = 0.506, *p* = 0.006; control: *r*_*s*_ = 0.406, *p* = 0.015).

## Discussion

The current study provides the first evidence of abnormal sensitivity to the ILD and ITD in participants with ASD. Participants with ASD tended to exhibit poorer ILD and ITD sensitivity than the controls. Moreover, there was a significant diversity of ITD sensitivity in the ASD group, i.e., it contained a larger proportion of participants with poor ITD sensitivity than the control group. Such diversity was not observed in ILD sensitivity. The findings suggest that deficits in the processing of ILD and ITD cues underlies the degraded performance in horizontal sound localization in individuals with ASD.

The overall results indicate that participants with ASD have poor sensitivity to the ILD and ITD relative to controls. This could be because participants with ASD have deficits in processing at the low level (e.g., the brainstem). Studies on albino cats, which exhibit abnormalities of MSO neurons, have shown poor ITD sensitivity at the level of the auditory midbrain^49^ and behavioral deficits in sound localization^50^. Anatomical studies indicate a decreased number of neurons and neural dysmorphology in the human SOC in ASD (e.g. LSO^17,18^ and MSO^16–19^), suggesting brainstem immaturity or arrested development^19,51^. Physiological studies showed prolonged wave III and V latencies and reduced amplitudes of BIC of ABRs in individuals with ASD^26–37^, indicating abnormal neural synchrony in the brainstem. Our finding of poor sensitivity to the ILD and ITD in the ASD group may be caused by these anatomical and neurophysiological abnormalities.

We observed a significant diversity in participants with ASD for the ITD thresholds but not for the ILD thresholds. This discrepancy in the threshold distribution between ILD and ITD indicates that the effect of ASD is different for neural mechanisms responsible respectively for the two cues, e.g., LSO and MSO. Our result for the ITD thresholds is in line with anatomical studies demonstrating a great variability in the morphology of the MSO neurons in ASD^16^. It can be regarded as psychophysical evidence supporting structural abnormalities in the brainstem reported in neuroanatomical studies of ASD.

Our results indicate that lower level (or cue-processing-level) mechanisms in the auditory pathway are implicated in the degraded performance of sound localization in individuals with ASD. Contributions of the higher level processes, however, remain possible. We observed significant correlations between the ILD and ITD thresholds in both the ASD and control groups, suggesting that ILD- and ITD-based lateralization performance depends on a common mechanism, at least partly. Similar correlation between the ILD and ITD thresholds was also found in the control group of the current study, and has also been reported in a past study with normal-hearing participants^52^. A candidate for the “common mechanism” is a higher level *sensory* process where information about ILD and ITD has been integrated, or a non-sensory *cognitive* process (it should be recalled that the current lateralization tasks employed a common a two-interval two-alternative forced-choice [2I-2AFC] procedure, which requires working memory). It should be reminded that participants were given common instruction (to indicate the laterality of the stimulus in the second interval relative to that in the first interval) in the ILD and ITD measurement. It is, however, difficult to account for the differences between the distributions of ITD and ILD thresholds, by such a common mechanism.

The ASD group contained a larger proportion of participants with poor ITD sensitivity than the control group, indicating the existence of subgroups. Several studies have classified individuals with ASD into subgroups based on cognitive-behavior characteristics^53^, neurophysiological characteristics^54^, and auditory skills^9,43,44^. The current finding on the diversity in ITD sensitivity adds a new insight into the pathogenesis and/or neurologic mechanism of ASD.

## Methods

### Participants

Thirty-one high-functioning adults with ASD and 40 control adults participated in the study. Two ASD adults and three control adults were excluded because of hearing loss ≥ 30-dB HL at one or more frequencies (*n* = 2 for ASD; *n* = 2 for control) or a full IQ score (assessed by WAIS-Ш) ≤ 70 (control: *n* = 1). Final participants included 29 high-functioning adults with ASD (aged 20–45 years) and 37 control adults (aged 20–37 years). They were statistically matched in age and the full IQ score (shown in Table 1).

The ASD participants were recruited from outpatient units of Karasuyama Hospital, Tokyo, Japan. The diagnosis of ASD was based on a consensus reached by two or three experienced psychiatrists according to the criteria of the Diagnosis and Statistical Manual of Mental Disorders (DSM-IV-TR and DSM-V). The control adults had no history of psychiatric illness or neurological disorders. Autism Spectrum Quotient (AQ) scores were obtained from all participants^55^. The AQ scores in the ASD group were higher than those in the control group (shown in Table 1).

The experiment was approved by the Ethical Committees of the NTT Communication Science Laboratories and was conducted in accordance with the Declaration of Helsinki. All participants signed written informed consent and were paid for their time.

### Apparatus and procedure

The stimuli for all tasks were generated digitally by a personal computer, transformed by an audio interface (Syntax, FirefaceUCX), and presented through headphones (Sennheiser, HDA200). All participants were tested in the same order on the following tasks: ILD threshold measurement, ITD threshold measurement, and auditory filter measurement. A 2I-2AFC task with feedback was used for all measurements. In the ILD and ITD threshold measurements, participants were required to indicate the laterality of the stimulus in the second interval relative to that in the first interval. In the auditory filter measurement, participants indicated the interval containing the pure-tone signal. Participants used a computer mouse and reported the answer by selecting from choices displayed on a computer monitor. All participants were given a couple of practice runs prior to data collection and were checked whether they could understand the tasks.

### ILD threshold measurement

Sensitivity to the ILD was measured in a lateralization discrimination experiment. The stimulus was a 400-ms bandpass-filtered noise (passband: 250–4000 Hz), including 50-ms raised-cosine onset and offset ramps. The 2I-2AFC procedure was used, in which the stimulus was presented bilaterally at a sound pressure level (SPL) of 65 dB (mean of the two ears) in each of the two intervals, separated by 200 ms. In the first interval, an ILD was presented favoring one side (i.e., higher in level), and in the second interval an ILD of the same level favored the other side, with the order randomized across trials. A two-down one-up stepping rule was used to track 70.7% correct performance^56^. The initial ILD was set to 3 dB. The step size was changed by 0.5 dB until the first reversal, by 0.25 dB until the third reversal, and by 0.125 dB thereafter. A run was terminated after 12 reversals, and the threshold was estimated as the mean of the threshold at the last eight reversals. Two threshold estimates were obtained, and a third estimate was obtained when the difference of the two thresholds exceeded 1 dB. The ILD threshold was taken as the mean of these two (or three) values.

### ITD threshold measurement

As in the ILD threshold measurement, a 400-ms bandpass-filtered noise with 65-dB SPL was used for the ITD threshold measurement, except that the onset and offset ramps had a duration of 100 ms. In the first interval, an ITD was presented favoring one side (i.e., advanced in time), and in the second interval an ITD of the same magnitude favored the other side, with the order randomized across trials. The two-down one-up stepping rule was also used for the ITD threshold measurement. The initial ITD was set to 40 μs. The step size was changed by a factor of 10^0.2^ until three reversals, and by a factor of 10^0.05^ thereafter. A run was terminated after 12 reversals, and the threshold was estimated as the geometric mean of the threshold at the last eight reversals. Two threshold estimates were obtained, and a third estimate was obtained when the difference of the two thresholds exceeded 5 μs. The ITD threshold was taken as the mean of these two (or three) values.

### Auditory filter measurement

Auditory-filter shapes were estimated by using a notched-noise masking method^45^. The signals were 200-ms pure tones with frequencies of 500, 1000, and 2000 Hz. The 400-ms notched noise was presented at 65-dB SPL. The signal and noise were ramped on and off with 20-ms raised-cosine ramps and were presented to the right ear. The detection threshold for the signal tone was measured at six relative notch widths g = Δf/fc: 0.01, 0.05, 0.1, 0.2, 0.3, and 0.4, where Δf is the notch width and fc is the signal frequency. Following the method reported by Santurette and Dau (2007)^57^, the lower and upper cut-off frequencies of the noise were set to fc (0.6 − g) and fc (1.4 + g), respectively. The 2I-2AFC procedure was used for the threshold measurement. In each trial, the two intervals contained the notched noise, and one randomly chosen interval also contained the tone signal. The two-down one-up stepping rule was also used, in which the initial presentation level of the signal was 75-dB SPL. The step size was changed by 8 dB until the first reversal, by 4 dB until the third reversal, by 2 dB until the fifth reversal, and by 1 dB thereafter. A run was terminated after 12 reversals, and the threshold was estimated as the mean of the threshold at the last eight reversals. Two threshold estimates were obtained, and a third estimate was obtained when the range of the two thresholds exceeded 10 dB. The mean of these two (or three) threshold value was calculated as a function of the relative notch width g for each subject. A rounded-exponential filter^46^ was fitted to the experimental data using a least-squares fit. The ERB was then derived from the parameters of the fitted filter.

Some participants (*n* = 12 for ASD: 41.4% of ASD; *n* = 17 for controls: 45.9% of controls) were assigned to the measurements for the auditory filter at the center frequencies of 500 and 2000 Hz, whereas others (*n* = 17 for ASD: 58.6% of ASD; *n* = 20 for controls: 54.1% of controls) were assigned to the measurements for the auditory filter at only one center frequency of 1000 Hz due to time constraints.

### Data analysis

All statistical analyses were conducted with SPSS software version 23 (SPSS Inc., Chicago, IL, USA). When computing the means and SDs (Table 4) or plotting a histogram (Fig. 1), a log transformation (to base 10) was applied to the ITD threshold data to make the data close to normal distribution.

## Data Availability

The datasets generated during and/or analyzed during the current study are available from the corresponding author on reasonable request.
